# A new method for estimating ore grade based on sample length weighting

**DOI:** 10.1038/s41598-023-33509-0

**Published:** 2023-04-17

**Authors:** Zhan-Ning Liu, Yang-Yang Deng, Rui Tian, Zhan-Hui Liu, Peng-Wei Zhang

**Affiliations:** 1 Anyang Institute of Technology, Anyang, Henan People’s Republic of China; 2 AnYang University, Anyang, Henan People’s Republic of China; 3 Harbin Center for Integrated Natural Resources Survey, China Geological Survey, Harbin, Heilongjiang People’s Republic of China

**Keywords:** Geology, Mineralogy

## Abstract

Estimation of ore grade is very important for the value evaluation of ore deposits, and it directly affects the development of mineral resources. To improve the accuracy of the inverse distance weighting (IDW) method in ore grade estimation and reduce the smoothing effect of the IDW method in grade estimation, the weight calculation method involved in the IDW method was improved. The length parameter of the ore sample was used to calculate the weight of the IDW method. The length of the ore samples was used as a new factor of the weighting calculation. A new method of IDW integrated with sample length weighting (IDWW) was proposed. The grade estimation of Li, Al, and Fe in porcelain clay ore was used as a case study. A comparative protocol for grade estimation via the IDWW method was designed and implemented. The number of samples involved in the estimation, sample combination, sample grade distribution, and other factors affecting the grade estimation were considered in the experimental scheme. The grade estimation results of the IDWW and the IDW methods were used for comparative analysis of grades of the original and combined samples. The estimated results of the IDWW method were also compared with those of the IDW method. The deviation analysis of the estimated grade mainly included the minimum, maximum, mean, and coefficient of variation of the ore grade. The estimation effect of IDWW method was verified. The minimum deviations of the estimated grade of Li, Al, and Fe were between 9.129% and 59.554%. The maximum deviations were between 4.210 and 22.375%. The mean deviations were between − 1.068 and 7.187%. The deviations in the coefficient of variation were between 3.076 and 36.186%. The deviations in the maximum, minimum, mean, and coefficients of variation of the IDWW were consistent with those of the IDW, demonstrating the accuracy and stability of the IDWW method. The more the samples involved in the estimation, the greater the estimation deviations of IDW and IDWW methods. The estimated deviations of Li, Al, and Fe were affected by the shape of the grade distribution, when the same estimation parameters were used. The grade distribution pattern of the samples significantly influenced the grade estimation results. The IDWW method offers significant theoretical advantages and addresses the adverse effects of uneven sample lengths on the estimates. The IDWW method can effectively reduce the smoothing effect and improves the utilization efficiency of the original samples.

## Introduction

The importance of ore body grade estimation methods is evident due to their use as the premise in ore body value evaluation, mining design, and mining plan management^1^. Inverse Distance Weighting (IDW) method^2,3^ has been widely used^4,5^ as a deterministic estimation method^6,7^. At present, the related research directions of IDW methods can be divided into four types. First is the simple application of IDW method, which mainly involves the direct estimation. In this method, the estimated influencing factor parameters are based on the previous research results. The optimization of parameters mainly includes the power (p) value^8,9^ in the formula and the number of sample points involved in estimation (n value) or the neighborhood range (neighborhood radius) of the estimated points. Many studies take the p value of 2 as the typical value for estimation^10–12^. In this study, p with the value of 2 was used as the grade estimation parameter. In general, when IDW method is applied, it is often compared with Kriging method. Several comparison results show that IDW method and Kriging method have their own advantages^13,14^. However, some studies have shown that the estimation effect of IDW method is better than that of Kriging method^15,16^. Moreover, it has been found that Kriging method has higher smoothing effect than IDW method, and IDW method is superior to Kriging method in estimating smoothness. IDW is superior to Kriging method in minimum and maximum estimation^17^. Similarly, some studies have shown that Kriging method offers more advantages^18,19^. IDW exhibits the characteristics of low computing cost and flexible application^20^, and it has more estimation advantages in case of small samples^21^. Moreover, IDW method has been successfully applied to filter^22^, error correction^23^, etc. In this study, the simple IDW method was used for comparative analysis, and the Kriging method was not used. This is mainly attributed to the fact that there are not enough samples for variogram analysis in different directions, thus the application of ordinary Kriging method is limited.

Second method involves the research on parameter optimization of IDW estimation^24^. The influence of IDW parameter selection on estimation is discussed^25,26^. These factor parameters mainly include p value and n value (or domain radius). Research shows that IDW is very sensitive to weighting power (p value). The greater the weighting power, the smaller the effect that samples far from the prediction location have during estimation^27^. The research shows that when estimating rainfall data by IDW method, the impact radius of the optimal rainfall data was found to be in the range of 10–30 km, and the optimal p value varied from 0 to 5^28,29^. Moreover, some studies have analyzed the influence of the distribution shape of estimated samples data on the selection of estimation parameters^30^.

Third, IDW method is used in combination with other methods for estimation or other purposes. For example, in order to normalize the effects of terrain and land cover effects, a new method coupling random forest (RF) method and IDW was proposed, and named as RF-IDW. It was used in the estimation of temperature and precipitation over complex areas^31^. Furthermore, the Monte Carlo IDW method was proposed to interpolate nitrate concentration, and the sensitivity and accuracy of Monte Carlo IDW estimation were analyzed^32^. Based on the Least Squares Collocation (LSC) method and IDW method, a local gravity field modeling method, namely, IDW-LSC combining LSC and IDW was proposed to solve the limitations of single IDW modeling method in local gravity field modeling^33^. IDW method was also coupled with Contrast Radial Intensity (CRI) for image scene monitoring^34^. Moreover, compressed sensing was combined with IDW for Gauge Measured Rainfall interpolation to achieve better results over those obtained using pure IDW^35^. Some researches combined fractal method with IDW and proposed fractal IDW (MIDW) method^36^. In the MIDW method, weights for moving average are assigned based on the local scaling property of data, which is quantified by using a power-law function^37,38^.

Fourth method involves the improvement in the IDW method. These improvements mainly focus on the calculation of distance weight. The gradient plus-inverse distance squared (GIDS) method was proposed^39^. GIDS combines multiple linear regression and distance-weighting for weight calculation, which improves its effect in the estimation of climatic data. In order to reflect the individual characteristics of the spatial distribution of ore body grade and increase the estimation accuracy of IDW method, some studies have modified the weight calculation method to reflect the heterogeneity of the estimation space^40^. Similarly, in ore grade estimation, the Euclidean distance in distance weight is extended to Minkowski distance. In the study, the impact of more distance calculation types on ore grade estimation was analyzed^41^. The modified IDW estimation method was proposed. The proposed method did not require external drifts and exhibited the advantage of performing accurate Particulate Matter estimation through IDW weight correction^42^. The Augmented Inverse Distance Weighted method introduced the elevation parameter as the distance weight factor, and the elevation parameter became a variable for weight calculation, which led to the improvement in the estimation effect of rainfall data^43^. The Adjusting Inverse Distance (AIDW) method was proposed for use in unstructured mesh finite volume solutions. The AIDW method changes this distance to the area of isosceles triangle in the neighborhood of the sample point, which can better reflect the neighborhood range of the sample and make the weight calculation more reasonable^44^. Furthermore, an optimization of the IDW method was proposed, which used a new technique of choosing the nearest points during the estimation process (named as the growing radius) in the process of creating a Digital Terrain Model (DTM) of the seabed based on bathymetric data collected using a Multi Beam Echo Sounder (MBES)^45^. A new parameter (k) was used as Modified Inverse Distance Weight (MIDW) for building settlement prediction. The k value was considered according to the shielding relationship between the observation point and the prediction point, in order to improve the prediction accuracy^46^. A modified spatial estimation method called Adjusted Inverse Distance Weighted (AIDW) method was used to analyze meteorological data around the Islamic University, Bangladesh^47^. Notably, the AIDW is similar to MIDW in that the parameter k is added to the molecular position of distance weight; however, the value of k depends on the characteristics of the data. An active learning algorithm was used to solve regression problems based on inverse-distance weighting functions for selecting the feature vectors to query^48^. Although the data of these research cases are different, the improvement methods mainly involve the adjustment of the calculation method of distance weight, or consider the weight calculation of new factors.

In this study, the combination process for ore body samples was simplified, and the original samples were directly used to estimate the ore body grades. In order to reduce the sample combination for pretreatment, the sample length factor was considered in the weight calculation of IDW method to reduce the smoothing effect of the estimated grade caused by sample combination. At the same time, to reduce the influence of the spatial inhomogeneity of the sample grade, based on the IDW method, herein it was proposed to add the sample length as a grade weight (IDWW) for ore body grade estimation. The IDWW method also aided in optimization of IDW by enhancing weight calculation. This study theoretically improves the accuracy of grade estimation, reduces the number of grade combination steps, and improves the accuracy and efficiency of estimation.

## Study area

In this section, the analysis of the geological conditions of the study area is presented. The occurrence state of the ore body is explained. Further, construction of a three-dimensional (3D) model and block model of the clay ore body is presented.

### Geological features

The area where the research object is located is a magmatic rock distribution area, and only Quaternary (Q) loose layers are mainly distributed in some low-lying areas and valleys in the region. The area is mainly composed of residual slope accretion and alluvial–proluvial strata. The main lithology comprises brown–yellow, yellow, brown–red, gray–black humus, loamy clay, loamy soil, and gravel-bearing sandy soil.

The exploration line 16–12 in the mining area has exposed a small NE-trending compressive torsional fault (F3 fault), which is inclined to the west and has a dip angle of about 75°. Chloritization can be seen in the fault, and the 12-line drilling controls the fault breccia with a vertical width of 28 m. The folds in the region are mainly part of the complex Guyangzhai anticline. The axis of the complex anticline strikes nearly east–west, and the outcrop is about 40 km long. The core was intruded and destroyed by the Ganfang pluton in the Yanshan period.

The magmatic rocks in the study area are widely exposed and have complex lithology. They were medium-acid rock in the early stage of magmatic rock genesis and are present as a batholith. The rocks were also acid rock in the late stage of the magmatic rock. Alkaline dyke rocks were relatively developed in the later stage of the magmatic rock. In the mining area, the following are mainly exposed: the first intrusive granite (γ_5_^2−2a^), the second intrusive granite (γ_5_^2−2b^), and the third intrusive granite in the second stage of the early Yanshan period (γ_5_^2−2c^), and the first stage intrusive granite (γ_5_^3−1^) and the late Yanshan alkaline dyke (Vπ) in the late Yanshan period.

### Ore body characteristics

The No. 1 Ore in the mining area is irregular rock vein-like or elongated rock nodule-like, and the general trend is nearly north–south. The surface exposure width of the 16-24 exploration line to the east of the F3 fault in the mining area is about 150 m, and the drilling control trend is about 180 m long. The exposed width of the 12–0 exploration line west of the F3 fault is about 150 m, and it extends westward for 1000 m and thins to a pinch out near the 0 line. The 12th line control tendency is 100 m long. Line 0 controls the tendency at length of 30 m.

### Three-dimensional ore body modeling

**Figure 1:**
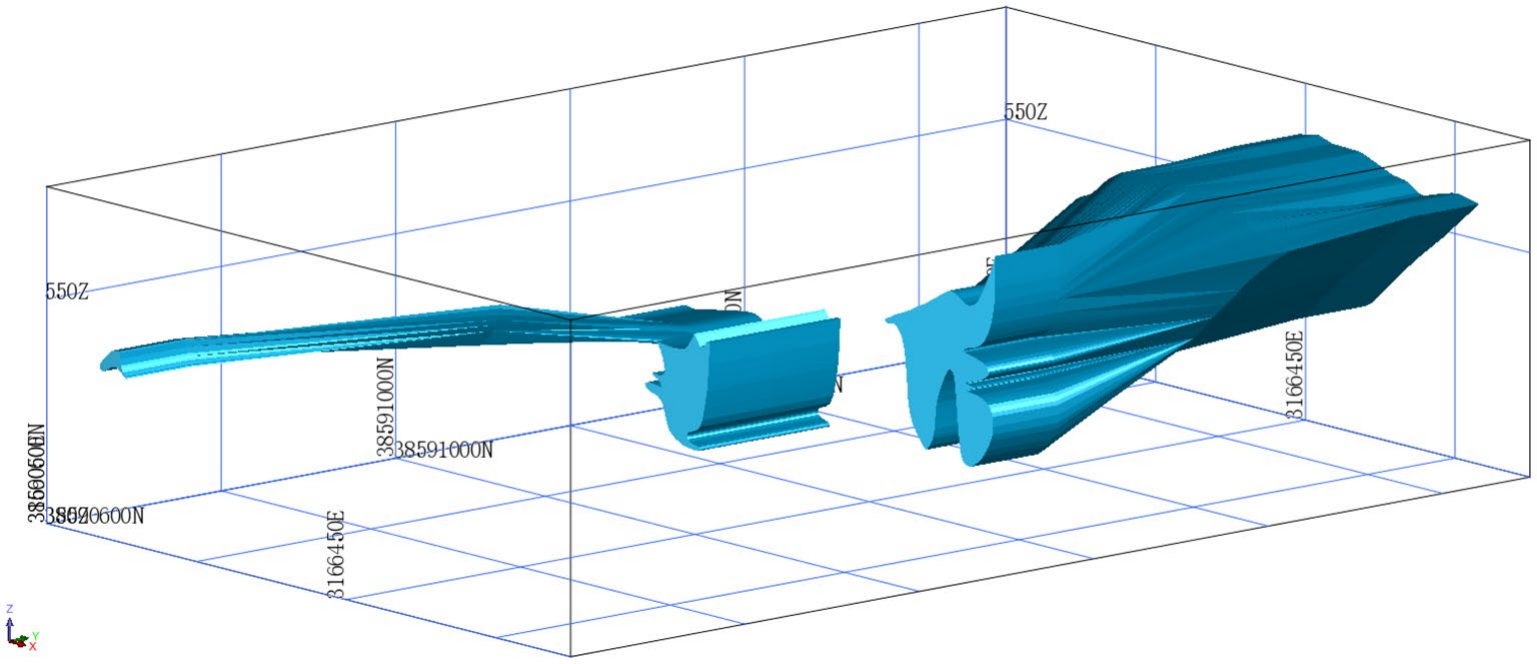
The 3D solid model of the ore body.

In this study, the original exploration line profile was used by employing the data format and coordinate conversion method; then, the two-dimensional (2D) ore body profile was used to establish a 3D solid model of the ore body. The model is shown in Fig. 1.

### Three-dimensional block model

**Figure 2:**
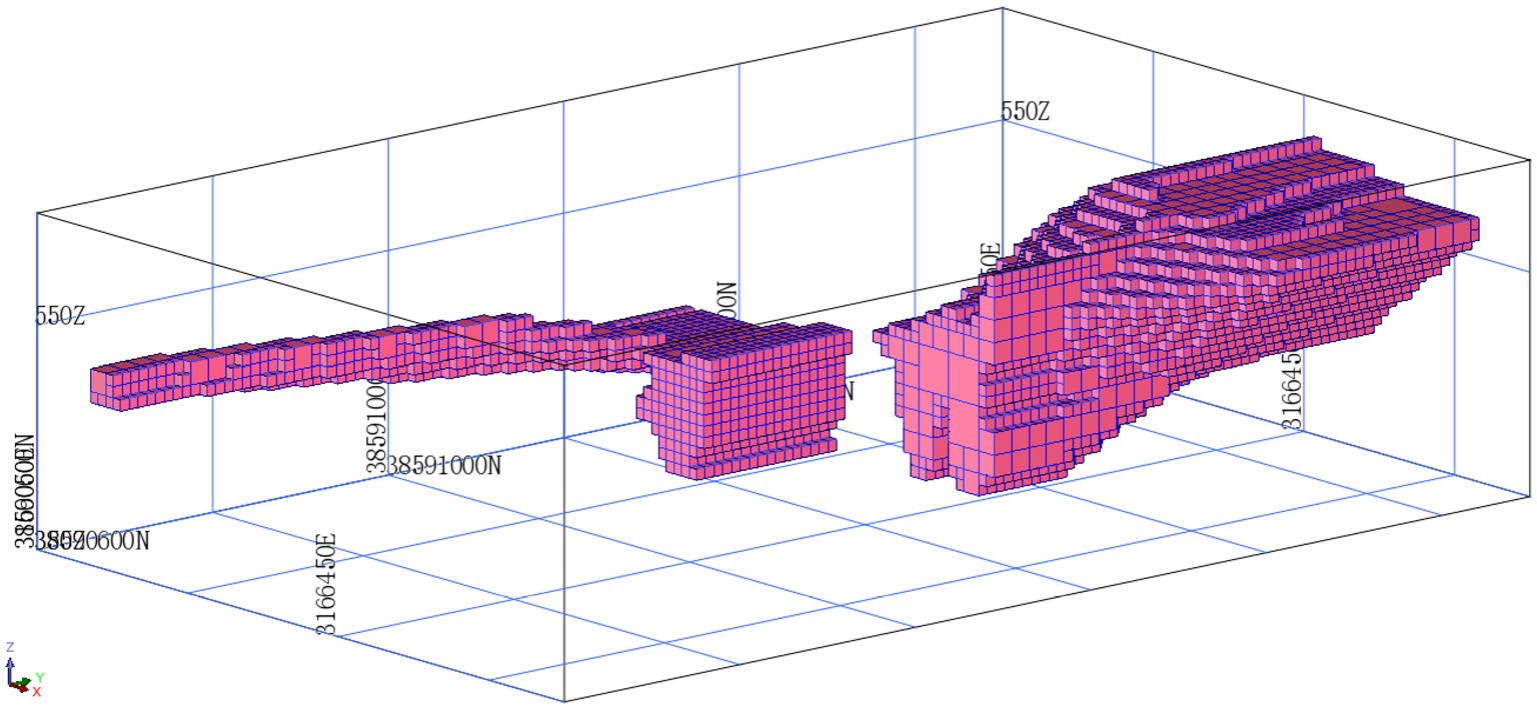
The block model.

The block model of the ore body was constructed based on the 3D solid model of the ore body. The block model of the ore body can be used to store estimated grade information. The size of the block unit is 10 × 10 × 10 m. The block model consists of a total of 12894 units. The block model is shown in Fig. 2. In this study, the 3D block model was output as a text file, which is convenient for MATLAB calculations.

## Research methods

### Theoretical analysis

#### The inverse distance weighting method

The IDW method is an interpolation method that is widely used for spatial information interpolation. It is also one of the most commonly used methods for the grade estimation of ore bodies. Its calculation formula is^49,50^ as follows:

1 $$P=\sum_{i=1}^{n}\frac{\frac{{M}_{i}}{{\left({d}_{i}\right)}^{p}}}{{\left(\frac{1}{{d}_{i}}\right)}^{p}}$$

In the abovementioned formula: *P* is the estimated ore grade; *n* is the number of samples involved in the grade estimation; *M*_*i*_ is the *i*-th grade value of the sample; *d*_*i*_ is the distance from the *i*-th sample to the estimated block; *p* is the power of the distance, and is generally a positive integer. The value of this *p* is chosen as 2^10–12^.

The IDW method takes the inverse of the distance between the sample and the block as the estimation of the weight. This weight is used to determine the contribution of the samples to the estimate. The closer the distance, the greater the impact. In contrast, the farther the distance becomes, the smaller the impact on the estimation result.

**Figure 3:**
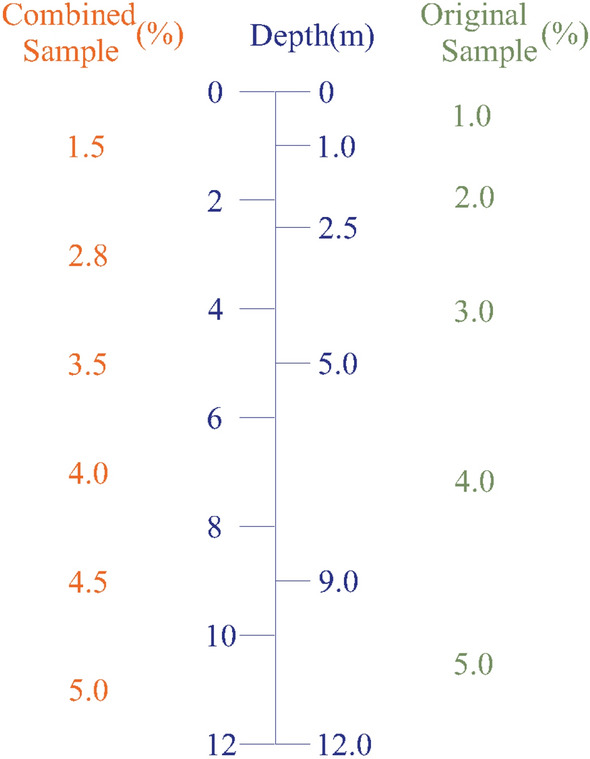
Schematic showing the sample combination.

Drilling equipment is used to extract rock samples from the interior of the earth during ore body exploration. Then, the rock samples are tested to analyze the mineral content in the rock. The mineral content determines the type of underground space occupied by the ore body^51^. However, the original samples obtained during exploration are not completely continuous. Sample analysis is also carried out section by section. As length of each assay sample is different, the assay data represent different sample lengths. For the assay data to represent the same sample length, the original samples need to be combined before grade estimation. The combined sample assay data represent the sample grade information of the same length. Its basic principle is shown in Fig. 3.

#### Sample combination method

The sample length combination method is shown in Fig. 3. Considering the combined sample length of 2 m as an example, the left side is a point generated at the middle position at a distance of 2 m, which is used to describe the combined value of the grade of the sample. On the right are the length and grade values of each original sample. The formula for calculating the sample combination is:

2 $${G}_{c}=\frac{\sum_{j=1}^{m}{G}_{j}*{L}_{j}}{\sum_{j=1}^{m}{L}_{j}} {L}_{c}\ge \sum_{j=1}^{m}{L}_{i}>0.5{L}_{c}$$

In the formula: *G*_*c*_ represents the combined sample grade; *G*_*j*_ represents the* j*-th sample grade within the length of the combined poplar; *L*_*c*_ represents the length of the combined sample; *L*_*j*_ represents the length of the* j*-th sample; *m* represents the number of samples participating in the combined sample.

#### The method of sample length weighting based on IDW (IDWW)

To reduce the number of sample processing steps during grade estimation, in this study, the original sample information was used to directly estimate the block grade while considering the influence of the sample length on the grade estimation. To this end, herein, an ore body grade estimation method is proposed by adding the sample length factor (IDWW). Formula (3) shows:

3 $$P=\sum_{i=1}^{n}\frac{\frac{{M}_{i}}{{\left({d}_{i}\right)}^{p}}*\frac{{L}_{i}}{L}*n}{{\left(\frac{1}{{d}_{i}}\right)}^{p}}$$

In the abovementioned formula: *L*_*i*_ is the length of the *i*-th sample; *L* represents the total length of *n* samples; The rest of the variables have the same meaning as those in formula (1).

### Statistics and combination of samples

**Figure 4:**
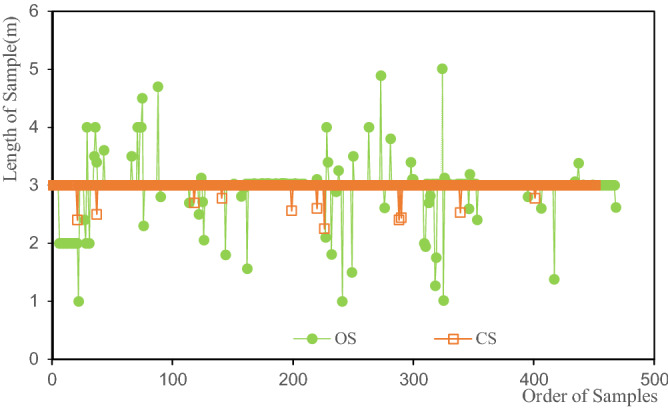
Statistics of the sample length.

The statistics of the sample length of the original samples (see Fig. 4) show that the sample lengths of the original samples are mostly concentrated at around 3 m. Therefore, the length of the combined sample was determined to be 3 m, and the minimum combined sample length was 75% of the combined length, that is 2.25 m. The statistics results of the combined sample length are presented in Table 1. The maximum length of the combined sample is 3 m and the minimum length is 2.25 m.

**Table 1:** Statistics of the OS and the CS.

Name	Li (%)		AL (%)		Fe (%)	
Sample type	Original	Combined	Original	Combined	Original	Combined
Minimum	0.2000	0.2067	8.9300	9.0452	0.2500	0.2500
Maximum	0.9400	0.9400	23.5700	23.5200	2.3000	2.1693
Mean	0.5151	0.5114	15.6412	15.5952	0.8990	0.8903
Variance	0.0148	0.0142	2.2967	1.7564	0.0701	0.0615
Standard deviation	0.1216	0.1194	1.5155	1.3253	0.2647	0.2481
Median	0.5200	0.5244	15.3800	15.3903	0.8500	0.8311
Kurtosis	0.0735	0.0207	1.8824	1.6363	1.6017	1.4490
Skewness	0.4336	0.4713	8.8185	9.6697	4.2676	3.7526
Variation coefficient	0.2360	0.2334	0.0969	0.0850	0.2944	0.2786

Basic statistic operations were performed on the original samples (OS) and the combined samples (CS). For convenience, the estimated grade was compared with the sample grade later in the study. Table 1 presents the Li, Al, and Fe statistics of the original and combined samples.

**Figure 5:**
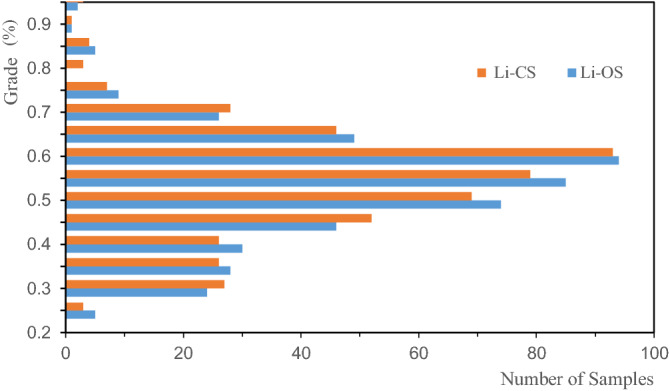
Distribution of Li in the original and combined samples.

**Figure 6:**
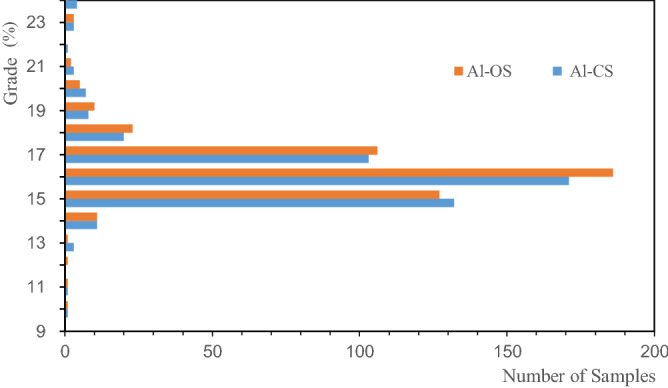
Distribution of Al in the original and combined samples.

**Figure 7:**
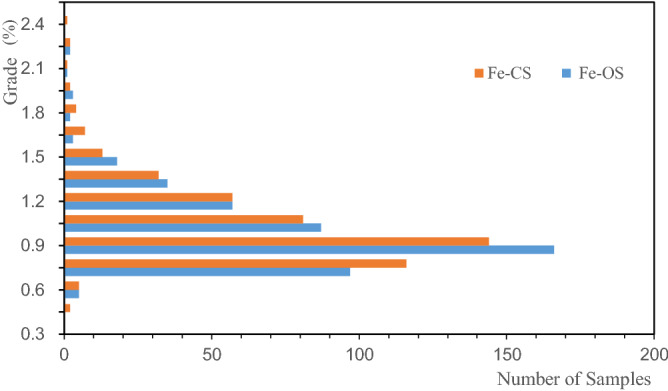
Distribution of Fe in the original and combined samples.

In order to analyze the influence of sample distribution on grade estimation, the histograms of the Li, Al, and Fe of the OS and the CS were drawn, as shown in Figs. 5, 6, 7. The figures illustrate that the distribution states of Li, Al, and Fe are different; the distribution of Al grade is concentrated, and that of the Li and Fe grade deviates from the low-grade range. The CS are in the lower grade range compared with the OS. Such a rule can also be reflected in the mean grades of the OS and CS presented in Table 1, and the mean grade of CS is lower than that of OS. It shows that when using CS for grade estimation, there is a risk of underestimating the ore body grade.

### Experimental design

The main objective of the experiment was to verify the estimation accuracy of the weighted estimation method of the sample length, and to analyze the influence of sample combination on the grade estimation. Data from the 3D ore body modeling, block modeling, and drilling were exported as text files in the pre-data preprocessing stage in this study. The text file mainly included block space position information and grade information of the original samples and the combined samples. MATLAB was used as the verification analysis tool.

**Figure 8:**
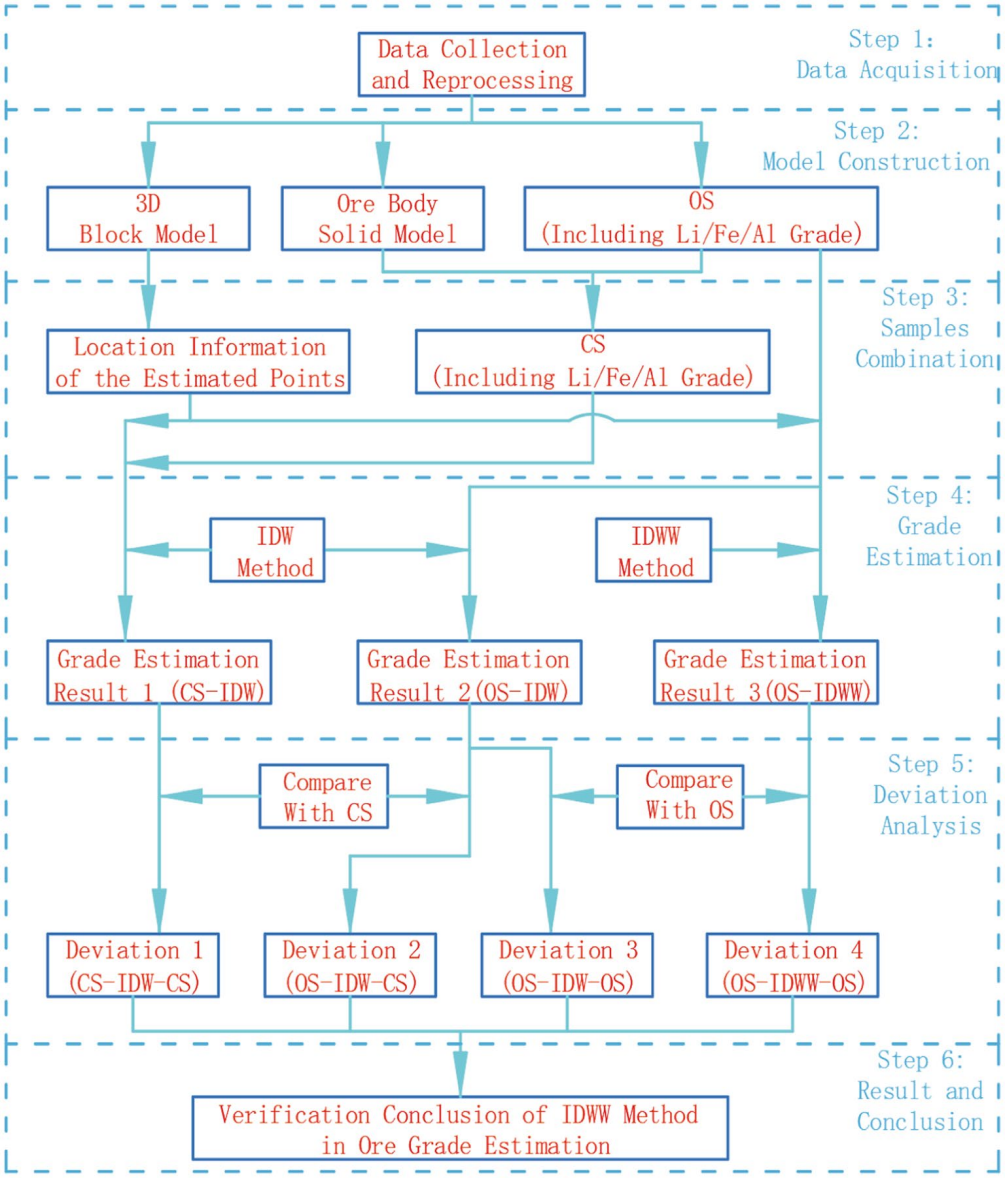
Study flow chart.

The main steps of the experiment are as follows:

1. Preprocessing of the experimental data: The OS data were extracted and the OS were combined. Data of the 3D block models, OS, and CS were converted to text format files. Non-essential information in the abovementioned text files was removed.
2. Determination of the type of ore body grade to be verified: The Li, Al, and Fe grades of the clay ore were used as the valuation verification grades.
3. Block grade estimation: Considering the Li, Al, and Fe grade information of the OS and the CS as the data source, the IDW method was used to estimate the grade of the block, and the grade estimation results were recorded as Result 1 and Result 2, respectively. Similarly, the grade information of Li, Al, and Fe was used as the data source, and the IDWW method was used to estimate the grade of the block, which was recorded as Result 3.
4. Calculation and statistics of the deviations: Result 1 and Result 3 were compared with the OS, and Result 2 was compared with the OS and the CS at the same time. The deviation of the minimum, maximum, mean, and coefficient of variation of the grade was calculated.
5. Analysis of results: The estimated effect of the IDWW method was analyzed. The effect of sample type on the estimated results was analyzed. The detailed process is presented in Fig. 8.

## Statistics and analysis of the estimation results

### Statistics of the estimation results

Statistics of the Li, Al, and Fe grade estimation results were studied. The statistic information includes the minimum, maximum, mean, variance, standard deviation, median, kurtosis, skewness, and coefficient of variation. The detailed results are presented in Tables 2, 3 and 4. OS represents the original samples and CS stands for combined samples. IDW stands for the inverse distance weighting method. IDWW stands for the inverse distance method that is based on sample length weighting. OS-IDW represents the result estimated by the IDW method with OS. CS-IDW represents the result estimated by the IDW method with CS. OS-IDWW represents the result estimated by the IDWW method with OS. The number of samples indicates the number of sample points for grade estimation. In this study, 3–7 samples were used to estimate the grade.

**Table 2:** Statistics of the Li grade estimation results.

Name of statistic	Method	Number of samples				
		3	4	5	6	7
Minimum	OS-IDW	0.2233	0.2391	0.2461	0.2523	0.2583
	CS-IDW	0.2256	0.2359	0.2344	0.2416	0.2461
	OS-IDWW	0.2200	0.2206	0.2313	0.2342	0.2427
Maximum	OS-IDW	0.8729	0.8345	0.8250	0.8056	0.7973
	CS-IDW	0.8492	0.8445	0.8162	0.7978	0.7887
	OS-IDWW	0.9004	0.8478	0.8279	0.7952	0.7779
Mean	OS-IDW	0.4765	0.4780	0.4781	0.4788	0.4789
	CS-IDW	0.4791	0.4835	0.4856	0.4852	0.4864
	OS-IDWW	0.4763	0.4777	0.4777	0.4785	0.4784
Variance	OS-IDW	0.0119	0.0115	0.0111	0.0106	0.0101
	CS-IDW	0.0112	0.0109	0.0108	0.0105	0.0104
	OS-IDWW	0.0119	0.0116	0.0111	0.0105	0.0101
Standard deviation	OS-IDW	0.1090	0.1074	0.1055	0.1029	0.1007
	CS-IDW	0.1058	0.1043	0.1037	0.1027	0.1022
	OS-IDWW	0.1091	0.1075	0.1055	0.1027	0.1004
Median	OS-IDW	0.4767	0.4800	0.4800	0.4785	0.4786
	CS-IDW	0.4833	0.4862	0.4871	0.4894	0.4968
	OS-IDWW	0.4767	0.4800	0.4799	0.4786	0.4786
Kurtosis	OS-IDW	−0.0789	−0.1253	−0.1442	−0.1935	−0.1657
	CS-IDW	−0.1592	−0.2667	−0.3058	−0.3102	−0.2813
	OS-IDWW	−0.0914	−0.1398	−0.1611	−0.2120	−0.1842
Skewness	OS-IDW	−0.3482	−0.4370	−0.4404	−0.4666	−0.4945
	CS-IDW	−0.3162	−0.3034	−0.2225	−0.2860	−0.2148
	OS-IDWW	−0.3584	−0.4490	−0.4519	−0.4765	−0.5001
Variation coefficient	OS-IDW	0.2287	0.2246	0.2206	0.2150	0.2102
	CS-IDW	0.2207	0.2157	0.2135	0.2116	0.2102
	OS-IDWW	0.2291	0.2251	0.2208	0.2145	0.2098

**Table 3:** Statistics of the Al grade estimation results.

Name of statistic	Method	Number of samples				
		3	4	5	6	7
Minimum	OS-IDW	10.881	11.183	11.819	12.012	12.483
	CS-IDW	10.999	11.208	11.808	12.019	12.486
	OS-IDWW	10.887	11.186	11.751	12.015	12.253
Maximum	OS-IDW	18.513	18.412	18.338	18.296	18.360
	CS-IDW	19.020	19.020	19.020	18.674	18.465
	OS-IDWW	18.513	18.643	18.549	18.629	18.844
Mean	OS-IDW	15.481	15.474	15.490	15.496	15.492
	CS-IDW	15.570	15.575	15.599	15.594	15.619
	OS-IDWW	15.489	15.478	15.492	15.499	15.505
Variance	OS-IDW	1.314	1.181	1.097	0.995	0.866
	CS-IDW	1.344	1.143	1.062	0.950	0.896
	OS-IDWW	1.337	1.194	1.110	0.998	0.919
Standard deviation	OS-IDW	1.146	1.087	1.047	0.998	0.930
	CS-IDW	1.159	1.069	1.030	0.975	0.947
	OS-IDWW	1.156	1.093	1.054	0.999	0.959
Median	OS-IDW	15.300	15.352	15.336	15.285	15.321
	CS-IDW	15.397	15.409	15.426	15.422	15.446
	OS-IDWW	15.322	15.349	15.337	15.285	15.321
Kurtosis	OS-IDW	0.0646	0.2384	0.3238	0.6286	0.7290
	CS-IDW	−0.071	−0.065	0.042	0.302	0.538
	OS-IDWW	0.0934	0.2425	0.3394	0.6112	0.8520
Skewness	OS-IDW	3.0789	3.2096	2.6931	2.4589	1.9065
	CS-IDW	2.665	3.231	2.885	2.441	1.900
	OS-IDWW	3.0280	3.1291	2.6486	2.4479	2.2796
Variation coefficient	OS-IDW	0.0741	0.0702	0.0676	0.0644	0.0601
	CS-IDW	0.074	0.069	0.066	0.063	0.061
	OS-IDWW	0.0746	0.0706	0.0680	0.0645	0.0618

**Table 4:** Statistics of the Fe grade estimation results.

Name of statistic	Method	Number of samples				
		3	4	5	6	7
Minimum	OS-IDW	0.318	0.330	0.370	0.379	0.388
	CS-IDW	0.2813	0.2977	0.3161	0.3420	0.3511
	OS-IDWW	0.325	0.348	0.386	0.392	0.399
Maximum	OS-IDW	1.853	1.800	1.880	1.840	1.793
	CS-IDW	1.8548	1.7946	1.8536	1.8336	1.7922
	OS-IDWW	1.853	1.800	1.880	1.840	1.793
Mean	OS-IDW	0.918	0.918	0.917	0.919	0.916
	CS-IDW	0.9152	0.9151	0.9200	0.9203	0.9221
	OS-IDWW	0.917	0.917	0.916	0.918	0.916
Variance	OS-IDW	0.065	0.062	0.060	0.059	0.058
	CS-IDW	0.0642	0.0569	0.0566	0.0551	0.0560
	OS-IDWW	0.065	0.062	0.060	0.058	0.058
Standard deviation	OS-IDW	0.255	0.250	0.245	0.243	0.240
	CS-IDW	0.2534	0.2385	0.2379	0.2347	0.2366
	OS-IDWW	0.255	0.248	0.244	0.242	0.241
Median	OS-IDW	0.850	0.855	0.854	0.853	0.850
	CS-IDW	0.8444	0.8375	0.8485	0.8446	0.8454
	OS-IDWW	0.850	0.851	0.854	0.851	0.847
Kurtosis	OS-IDW	1.0381	0.8793	0.9637	0.8807	0.8886
	CS-IDW	1.0896	0.9991	1.0568	0.9912	0.9807
	OS-IDWW	1.0377	0.8833	0.9694	0.8887	0.8927
Skewness	OS-IDW	1.0374	0.1720	0.6645	0.2776	0.3602
	CS-IDW	1.1918	0.6397	0.9704	0.6401	0.5164
	OS-IDWW	1.0304	0.2060	0.7072	0.3092	0.3358
Variation coefficient	OS-IDW	0.2778	0.2719	0.2673	0.2640	0.2618
	CS-IDW	0.2768	0.2606	0.2586	0.2551	0.2566
	OS-IDWW	0.2779	0.2708	0.2666	0.2634	0.2631

### Analysis of the estimated results

In this study, the estimated grades of Li, Al, and Fe in the magnetite ore body were compared with the grades of the samples, and the deviation between the estimated grades and the minimum, maximum, mean, and variation coefficients of the grades of the OS and CS was calculated. The evaluation effect was analyzed by using grade estimation deviation. Furthermore, the estimation characteristics of the IDWW method were analyzed and the influence of sample type and grade distribution characteristics on the estimation results was investigated by exploring the laws related to the IDW and IDW grade estimation. The deviation is calculated as: deviation = (estimation grade − sample grade)/sample grade × 100%. The first part of the estimation in the CS-IDW-OS represents the sample type, the second part represents the estimation method, and the third part refers to which sample type the deviation is from. The other representations are similar to this.

#### Li grade deviation analysis

**Figure 9:**
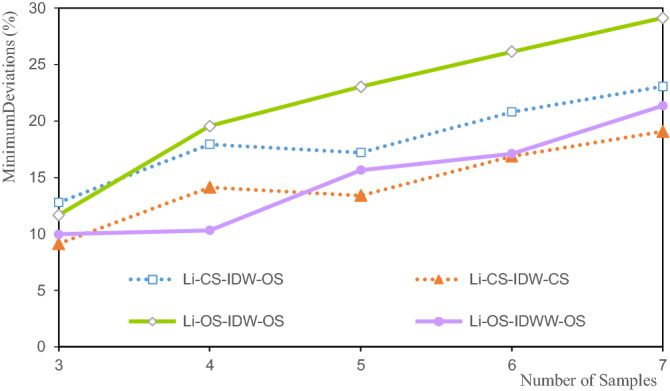
Deviation of the Li minimum grade.

**Figure 10:**
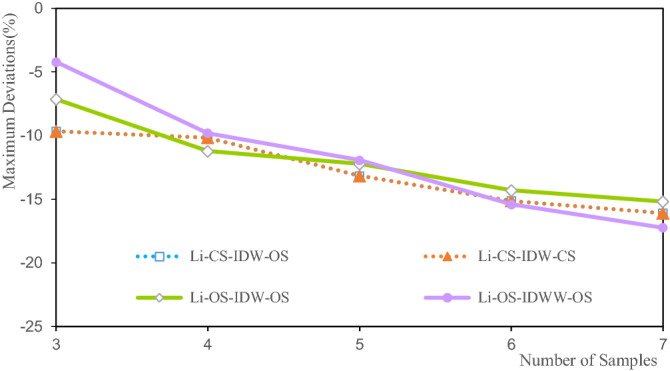
Deviation of the Li maximum grade.

**Figure 11:**
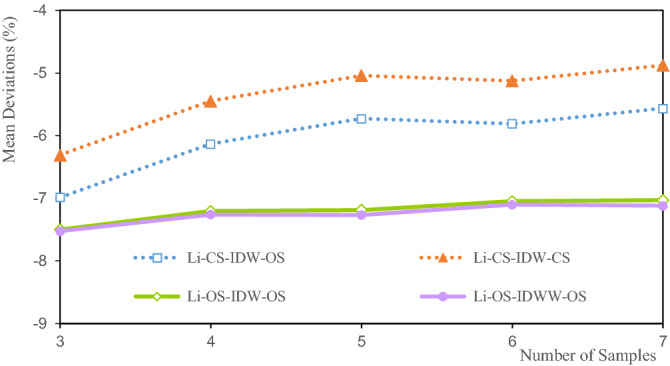
Deviation of the Li average grade.

**Figure 12:**
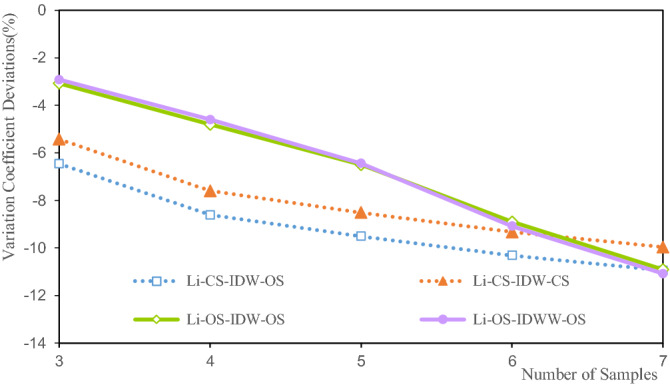
Deviation of the variation coefficient of the Li grade.

Figures 9, 10, 11 and 12 show the estimated deviation of the minimum, maximum, mean, and variation coefficient of the Li estimated grade, respectively. The horizontal axis represents the number of samples involved in the estimation and the vertical axis represents the deviation.

Figure 9 shows that the deviations of the estimated minimum grade are between 9.13% and 26.14%. The deviation of CS-IDW-CS is the smallest when there are three samples, and the deviation of OS-IDW-OS is the largest when there are seven samples. The deviations of the minimum of the Li estimated grade increase with the increase in the number of samples involved in the estimation, indicating that the more the number of samples involved in the estimation of the grade, the stronger the smoothness of the estimation result. The deviations of the Li estimated grade minima for CS-IDW-OS and CS-IDW-CS are in strong agreement, as shown by the two dashed lines in Fig. 9. The deviations of CS-IDW-OS are greater than those of CS-IDW-CS. The deviation of the Li estimated grade minimum of OS-IDW-OS is slightly larger than that of the other minima. An increase in the deviation of the estimated grade minimum indicates that the estimated minimum grade is closer to the mean grade. The OS-IDWW is more scientific in theory compared with OS-IDW. Moreover, the deviation of the Li estimated minimum grade with IDWW is relatively small.

The deviations of the Li estimated grade maximum range from − 4.6% to − 17.24%. Among them, the OS-IDWW-OS with three samples, participates in the estimation to achieve the smallest deviation of the maximum grade of − 4.21%, and the maximum deviation with the largest grade is 17.24% when seven samples participate in the estimation. The maximum deviations increase with the increase in the number of samples involved in the estimation. This is attributed to the fact that the larger the number of samples, the smoother the estimation result. Clearly, the deviations of CS-IDW-OS and CS-IDW-CS are highly consistent, and their deviations basically coincide with the maximum deviations of the Li grade. It shows that the maximum values of Li grade of the OS and the CS are close. The OS-IDW-OS and OS-IDWW-OS deviations are consistent. They are close to the deviations of CS-IDW-OS and CS-IDW-CS. The accuracy and stability of IDWW are demonstrated by the estimation results.

The mean deviations of the Li grade are between − 5.1% and − 7.5% (Fig. 11). The mean deviation of CS-IDW-CS is the smallest at seven samples, at 5.1%. The mean deviation of OS-IDWW-OS at three samples is the largest (− 75%). The mean deviations of the Li grades for CS-IDW-CS and CS-IDW-OS are highly consistent. The mean deviations of the Li grade decrease with the increase in the number of samples. The mean deviations of OS-IDW-OS and OS-IDWW-OS are highly consistent and their deviations decrease slowly with the increase in the estimated number of samples. Theoretically, the more the samples involved in the estimation, the smoother the estimation result. Smoothing indicates that the estimated interior grade of the ore body tends to be more average. Owing to the influence of the spatial morphology of ore bodies and the uneven distribution of samples, the same sample can have different effects on the surrounding block units. The distance from the sample to the different blocks is not exactly the same. Therefore, it is difficult for the statistical results of the estimated grade of the ore body to be exactly the same as the statistical results of the sample grade. The mean deviation of the estimated Li grades shown in Fig. 11 can only explain the variation trend between the estimated grades and the samples to a certain extent. Alternatively, this study demonstrates the relative accuracy of the estimated grade mean and the stability of the IDWW method.

Figure 12 shows that the variation coefficient deviations of the Li estimated grade are between − 2.92% and − 11.08%. When there are three samples, OS-IDWW-OS shows the smallest variation coefficient deviation, at − 2.92%, and when there are seven samples, the deviation of the variation coefficient is the largest, at 11.08%. The deviations of the variation coefficient of the Li grade show a decreasing trend with the increase in the number of samples. The variation coefficient reflects the dispersion of the samples to a certain extent. The increase in the deviations of the variation coefficient indicates that the difference between the variation coefficient of the estimated grade and the variation coefficient of the sample grade is enhanced. The essence of this enhancement is the increase in the smoothness of the valuation grade.

Figure 12 shows that CS-IDW-OS and CS-IDW-CS have a high degree of consistency in the deviation of the grade variation coefficient. The deviations of the coefficients of variation of OS-IDW-OS and OS-IDWW-OS are highly consistent, and they exhibit the same trend as the deviations of the coefficients of variation of the grades of CS-IDW-CS and CS-IDW-OS.

#### Al grade deviation analysis

**Figure 13:**
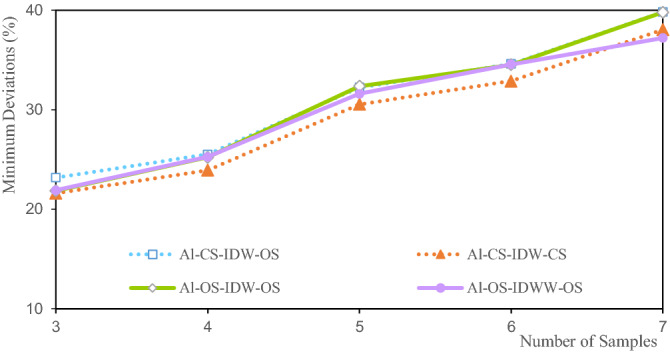
Deviation of Al minimum grade.

Figure 13 shows that the minimum deviation of the estimated Al grade is between 21.60 and 39.82%. The minimum deviation of CS-IDW-CS is 21.60% at three samples. The minimum deviation of OS-IDW-OS is 39.82% at seven samples. The minimum deviations of the Al estimated grade increase with the increase in the number of samples, indicating that the more the number of samples, the stronger the smoothness of the estimated result. There is a high degree of agreement between the estimated grade minimum deviations for the Al estimates across all estimates. The minimum deviations increase with the increase in the number of samples, indicating that the minimum grade is close to the average value of the Al grade. The variation of the Al grade minimum deviations obtained from the different estimation methods is relatively stable. The variation interval of the minimum deviations of the same samples is within 5%. The deviations of the Al estimated grade minimum show that IDWW has a similar estimation accuracy and stability compared with other methods.

**Figure 14:**
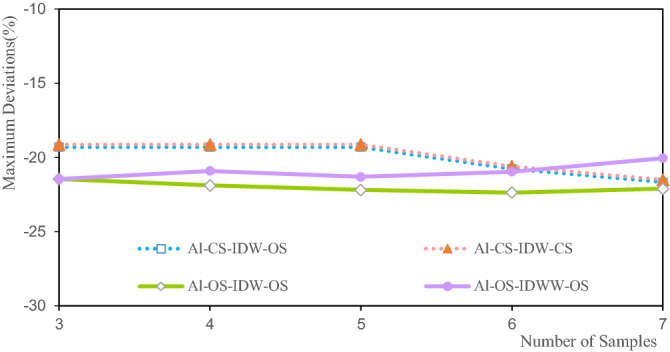
Deviation of the Al maximum grade.

Figure 14 shows that the maximum deviations of the estimated Al grade are between 19.13 and 22.38%. When there are three samples, the maximum deviation of CS-IDW-CS is 19.13%, and when there are seven samples, the OS-IDW-OS maximum deviation is 22.38%. Figure 14 illustrates that the maximum deviations of the Al grades estimated by CS-IDW-CS and CS-IDW-OS are completely consistent, and the variation trend first stabilizes and then increases with the increase in the number of samples. The maximum deviations of the Al grades as estimated by OS-IDW-OS do not change significantly. The maximum deviations of the Al grades as estimated by OS-IDWW-OS tend to decrease slowly. The estimation results of OS-IDWW-OS are generally consistent with the estimation results of the other methods, and IDWW estimation offers both accuracy and stability.

**Figure 15:**
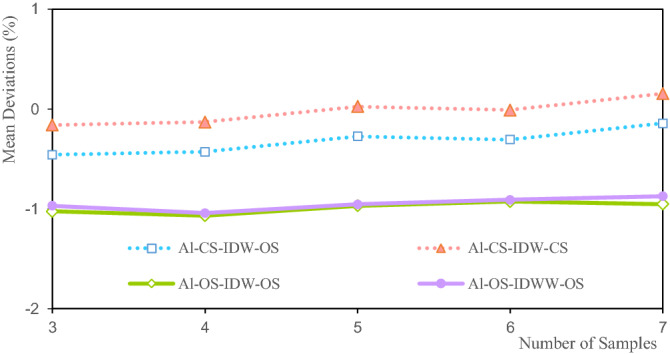
Deviation of the Al average grade.

Figure 15 shows that the mean deviations of the Al grade are between 0.154 and − 1.068%. The mean deviation of the Al grade estimated by CS-IDW-CS at seven samples is 0.154%. The mean deviation of the Al grade estimated by OS-IDW-OS at four samples is 1.068%. Figure 15 shows that the mean deviation of the Al grade estimated by CS-IDW-OS and CS-IDW-CS is highly consistent. Furthermore, the mean deviations of the Al grade show a slow decline with the increase in the number of samples. The mean deviations of OS-IDW-OS and OS-IDWW-OS are highly consistent, and the deviations show a slow downward trend with the increase in the number of samples. The variation trend of the mean deviations of the Al grade shown in Fig. 15 is basically the same as that of the mean deviations of the Li grade shown in Fig. 11. However, the mean deviations of the grade are quite different, which further shows that the influence of the sample grade distribution on the estimation results of IDW and IDWW methods is very significant.

**Figure 16:**
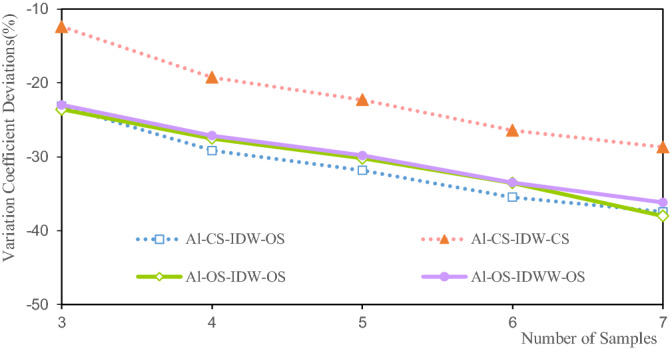
Deviation of the variation coefficient of the Al grade.

Figure 16 shows that the variation coefficient deviations of the Al grade are between 12 and 37.6%. Among them, when there are three samples, the variation coefficient deviation of the CS-IDW-CS grade is 12.5%. The variation coefficient deviation of the OS-IDW-OS grade is 37.6% at seven samples. The variation coefficient deviations of the Al grade estimated by IDW and IDWW exhibit a high degree of consistency, and the deviations increase with the increase in the number of samples, as shown in Fig. 16. The increase in the deviations of the variation coefficients indicates that the smoothness of the valuation grade increases with the increase in the number of samples. The variation coefficient deviations of OS-IDWW-OS exhibit the same trend as the deviations of CS-IDW-CS, CS-IDW-OS, and OS-IDW-OS. Furthermore, the deviations of OS-IDWW-OS are close to CS-IDW-OS and OS-IDW-OS, which also verifies the reliability and stability of the IDWW estimation.

#### Fe grade deviation analysis

**Figure 17:**
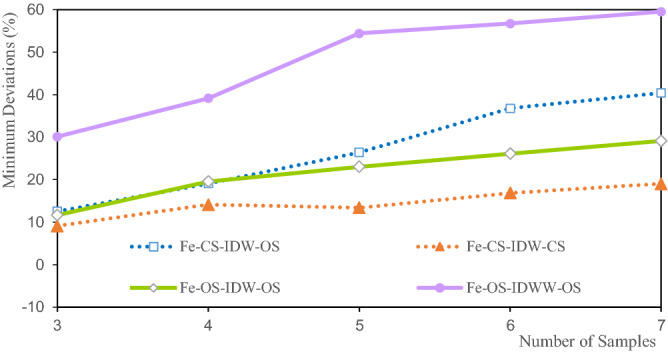
Deviation of the Fe minimum grade.

Figure 17 shows that the minimum deviations of the Fe estimated grade are between 12.533 and 59.554%. The minimum deviation of the Fe grade of CS-IDW-CS is 12.533% at three samples. The grade minimum deviation of OS-IDWW-OS is 59.554% at seven samples. The minimum deviations of the Fe estimated grades increase with the increase in the number of samples. It shows that the more the number of samples involved in the estimation, the stronger the smoothness of the estimation result. This is consistent with the variation trend of the estimated grade minimum deviations of Li and Al. The minimum deviations of the OS-IDWW-OS grade have the same relative change trend as those of OS-IDW-OS, CS-IDW-OS, and CS-IDW-CS. The minimum deviations of the Fe grade of OS-IDWW-OS are the largest. However, this cannot explain the poor estimation effect of the IDWW method. It is believed that the results are mainly affected by the spatial location of the samples and the distribution of the sample grades. The minimum deviations of the Fe grade of IDWW are consistent with the minimum deviations of the Fe grade of IDW, which demonstrates the feasibility and stability of the IDWW method

**Figure 18:**
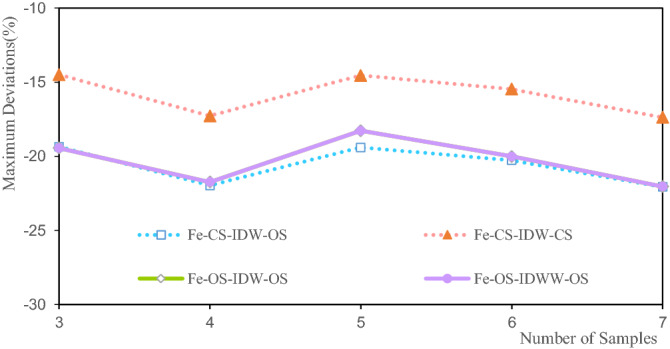
Deviation of the Fe maximum grade.

Figure 18 shows that the maximum deviations of the Fe estimated grade are between 14.497 and 22.077%. Among them, the maximum deviation of the Fe grade of CS-IDW-CS is 14.497% when there are three samples. The maximum deviation of the Fe grade of CS-IDW-OS at seven samples is 22.077%. With the increase in the number of samples, the maximum deviations of the estimated Fe grade first increase, then decrease, and finally increase again. Furthermore, the overall trend is an increasing one. The maximum deviations of the Fe grade exhibit a high consistency with the different methods and samples. It also shows that the IDWW estimation is accurate and reliable.

**Figure 19:**
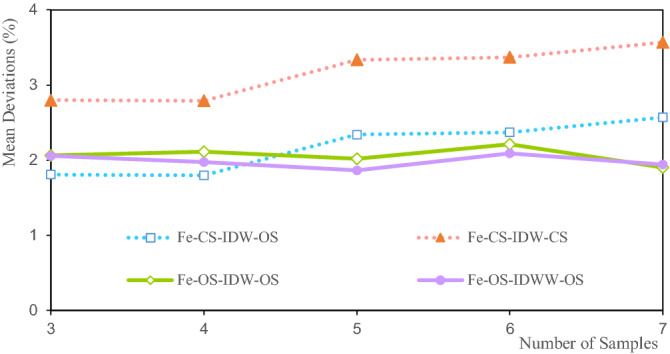
Deviation of Fe average grade.

Figure 19 shows that the mean deviations of the Fe grade are between 1.808 and 3.568%. Among them, the mean deviation of CS-IDW-OS is 1.808% when there are three samples. The grade mean deviation of CS-IDW-OS is 3.568% at seven samples. The mean deviation of the Fe grade is within a small range. The mean deviations of CS-IDW-OS and CS-IDW-CS increase with the increase in the number of samples. The mean deviations of OS-IDW-OS and OS-IDWW-OS are highly consistent and they show a slow downward trend with the increase in the number of samples. Theoretically, the more the number of samples involved in the estimation, the smoother the estimation result. This smoothing indicates that the estimated grade within the ore body tends to be closer to the grade mean. The average grades of CS-IDW-OS and CS-IDW-CS show an upward trend that is influenced by the combination of samples. The combination of samples changes the spatial distribution of the Fe grades, which eventually leads to changes in the mean deviations of the estimated Fe grades. Therefore, it can be inferred that the sample grade distribution exhibits a significant impact on the estimation results of the ore body grade. Therefore, it can also be inferred that the spatial distribution of sample grades has a significant impact on the estimation results of the ore body grade.

**Figure 20:**
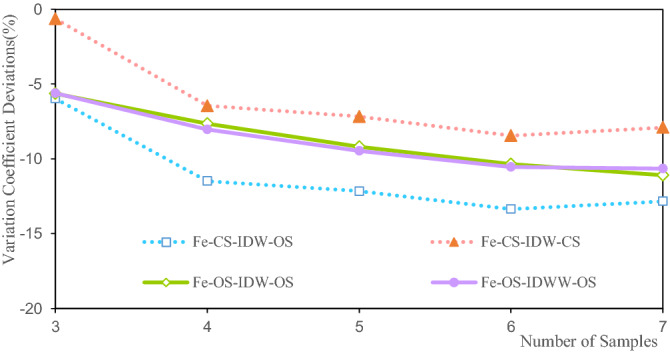
Deviation of the variation coefficient of the Fe grade.

The variation coefficient deviations of the estimated Fe grade are between 0.643 and 13.363%, as shown in Fig. 20. The variation coefficient deviation of the estimated grade of the CS-IDW-CS is 0.8% at three samples. The variation coefficient deviation of the estimated grade of CS-IDW-OS is 13.5% at six samples. The variation coefficient deviations of the Fe estimated grade are consistent based on the CS and OS using the IDW and IDWW methods, and the deviations increase with the increase in the number of samples, as shown in Fig. 20. The estimated Fe grade variation coefficient of IDWW is close to the Fe grade variation coefficient obtained by IDW. The variation coefficient deviations of the grade are consistent, which demonstrates the feasibility and stability of the IDWW method.

## Discussion

The minimum deviations of the estimated grades of Li, Al, and Fe shown in Figs. 9, 13 and 17 indicate that the minimum deviations increase with the increase in the number of samples involved in the evaluation. The minimum deviations are between 9.129 and 59.554%, and the overall minimum deviations are relatively large. The maximum deviations of the grades also increase with the increase in the number of samples involved in evaluating the maximum deviations of the estimated grades of Li, Al, and Fe shown in Figs. 10, 14 and 18. The maximum deviations are between 4.210 and 22.375%, and the overall maximum deviations are relatively large. These are mainly determined by the distribution of sample grades. The number of samples with smaller or larger grades is relatively small. During the grade estimation process, at least three samples were used in the grade estimation. Therefore, the estimated minimum grade was expected to be greater than the minimum grade of the sample and the estimated maximum grade was expected to be less than the maximum grade of the sample. Theoretically, the more the number of samples involved in the estimation, the closer the estimated minimum or maximum grade to the mean grade. Furthermore, there are greater deviations of the estimated minimum or maximum grade when more samples are involved in the estimation. In the grade estimation stage, the combination of samples causes the smoothing effect of the grade before the estimation, and the smoothing effect caused by IDW makes the estimation effect smoother. The maximum and minimum grade estimation deviations of the IDWW are consistent with those of the IDW, demonstrating the accuracy and stability of the IDWW method.

Figures 11, 15 and 19 show that the mean deviation of the estimated grade is between − 1.068 and 7.187%. The estimated mean grades of Li and Al are greater than the mean sample grades. The estimated mean grade of Fe is slightly smaller than the mean grade of the samples. The grade data for Li, Al, and Fe are obtained from the same exploration project, and from different assay grades of the same sample. Therefore, the grade distribution state of Li, Al, and Fe affects the mean deviation of the estimated grade, which cause the mean deviations of the Li, Al, and Fe grades to be different from each other. Moreover, under the same estimation parameters, the estimated deviations can only be used to evaluate the estimation accuracy to a certain extent. Furthermore, the estimated deviations are also affected by the shape of the grade distribution. The estimated results of IDW and IDWW are generally close when the same estimated sample and estimation parameters are used. The feasibility and stability of the IDWW method are demonstrated again.

Figures 11, 16 and 20 show that the deviations in the variation coefficient of the estimated grade are between 3.076 and 36.186%. Among them, the variation coefficient deviations of the estimated Li and Fe grades are significantly larger than those of the estimated Fe grades. The grade variation coefficient can reflect the dispersion of the grade distribution. The larger the coefficient of variation, the more dispersed the distribution of the statistical objects. The variation coefficients of the estimated grades of Li, Al, and Fe are all smaller than those of the sample grades. Therefore, the estimated grade distribution is more concentrated than the grade distribution of the samples. In other words, the estimated grades are close to the mean grade. It shows that the estimated grades assessed by IDW and IDWW methods exhibit a significant smoothing effect. However, the variation coefficient deviation of OS-IDWW-OS is generally smaller than that of CS-IDW-OS. This result shows that the IDWW method can effectively reduce the smoothing effect caused by sample combination and the estimation process.

Figure 4 exhibits the length distribution of the original samples, revealing that the length of the original samples is mostly about 3 m. Furthermore, the samples with a sample length of about 3 m account for the majority. Therefore, the estimated deviations from IDW and IDWW methods are closer when the estimation is carried out by using the data of the original samples, making the accuracy performance of IDWW not particularly significant. However, the IDWW method offers significant theoretical advantages because it addresses the adverse effects of uneven sample lengths on the estimates. The IDWW method also eliminates the effects of combining samples and reduces the smoothing effect caused by the combination of samples. The IDWW method improves the utilization efficiency of the original samples (Supplementary Information).

## Conclusion

In this study, the IDWW method was proposed, which considered the sampling length of the sample as the weight factor of grade estimation that reduced the smoothing effect caused by the sample grade combination process and sample combination. The IDWW method theoretically improved the estimation effect of ore grade. Li, Al, and Fe grades from porcelain clay were used as an experimental case of ore grade estimation. The reliability and accuracy of the IDWW method were verified by comparing the grade estimation results of the IDWW method with the IDW method. Moreover, the effects of sample quantity, sample grade combination, and sample grade distribution on ore grade estimation were also systematically analyzed. The results of this study are as follows:

1. The minimum deviations of the estimated grades of Li, Al, and Fe increased with the increase in the number of samples involved in evaluation. The minimum deviations were between 9.129 and 59.554%. The maximum deviations of the grades also increased with the increase in the number of samples. The maximum deviations were between 4.210 and 22.375%. The mean deviation of the estimated grade of Li, Al, and Fe were between − 1.068% and 7.187%. The deviations in the coefficient of variation of the estimated grade of Li, Al, and Fe were between 3.076 and 36.186%. The maximum, minimum, mean, and coefficients of variation grade estimation deviations of the IDWW were consistent with those of the IDW method, demonstrating the accuracy and stability of the IDWW method.
2. The number of samples involved in the estimation exhibited a direct impact on the estimated grade deviations. The more the samples involved in the estimation, the greater the estimation deviations of IDW and IDWW methods. The estimated deviations of Li, Al, and Fe were affected by the shape of the grade distribution, when the other estimated parameters remained unchanged. The distribution pattern of the spatial grade information of the samples significantly influenced the grade estimation results.
3. The estimated grades estimated by IDW and IDWW methods exhibited a significant smoothing effect. The IDWW method offered significant theoretical advantages because it addressed the adverse effects of uneven sample lengths on the estimates. The IDWW method was able to effectively reduce the smoothing effect caused by sample combination and the estimation process. Moreover, the IDWW method improved the utilization efficiency of the original samples.
4. The number of times each ore sample was used for the grade estimation was not exactly the same. Therefore, the statistical results of the estimated grade and the samples grade were not completely consistent. It was speculated that there might be deviations between the sample grades and the estimated grades. These deviations were not entirely due to the estimation method. The study also showed that the spatial location of sample sampling and the spatial shape of ore body were factors that affected the estimation of ore grade. Undeniably, a lot more systematic explorations are further demanded to analyze the impact of the spatial location of sample sampling and the spatial shape of ore body on the estimation of ore grade, which will be pursued in the future.

## Supplementary Information

Supplementary Information.

## Data Availability

All data generated or analyzed during this study are included in this published article and its supplementary information files.
